# Variations in Helicobacter pylori Cytotoxin-Associated Genes and Their Influence in Progression to Gastric Cancer: Implications for Prevention

**DOI:** 10.1371/journal.pone.0029605

**Published:** 2012-01-03

**Authors:** Cosmeri Rizzato, Javier Torres, Martyn Plummer, Nubia Muñoz, Silvia Franceschi, Margarita Camorlinga-Ponce, Ezequiel M. Fuentes-Pananá, Federico Canzian, Ikuko Kato

**Affiliations:** 1 German Cancer Research Center (DKFZ), Heidelberg, Germany; 2 Unidad de Investigacion en Enfermedades Infecciosas, Unidad Médica de Alta Especialidad (UMAE) Pediatria, Instituto Mexicano del Seguro Social (IMSS), Mexico City, Mexico; 3 International Agency for Research on Cancer (IARC), Lyon, France; 4 National Cancer Institute of Colombia, Bogota, Colombia; 5 Karmanos Cancer Institute, Wayne State University, Detroit, Michigan, United States of America; National Cancer Center, Japan

## Abstract

*Helicobacter pylori* (HP) is a bacterium that colonizes the human stomach and can establish a long-term infection of the gastric mucosa. Persistent Hp infection often induces gastritis and is associated with the development of peptic ulcer disease, atrophic gastritis, and gastric adenocarcinoma. Virulent HP isolates harbor the cag (cytotoxin-associated genes) pathogenicity island (cagPAI), a 40 kb stretch of DNA that encodes components of a type IV secretion system (T4SS). This T4SS forms a pilus for the injection of virulence factors into host target cells, such as the CagA oncoprotein. We analyzed the genetic variability in *cagA* and other selected genes of the HP cagPAI (*cagC*, *cagE*, *cagL*, *cagT*, *cagV* and *cag Gamma*) using DNA extracted from frozen gastric biopsies or from clinical isolates. Study subjects were 95 cagA+ patients that were histologically diagnosed with chronic gastritis or gastric cancer in Venezuela and Mexico, areas with high prevalence of Hp infection. Sequencing reactions were carried out by both Sanger and next-generation pyrosequencing (454 Roche) methods. We found a total of 381 variants with unambiguous calls observed in at least 10% of the originally tested samples and reference strains. We compared the frequencies of these genetic variants between gastric cancer and chronic gastritis cases. Twenty-six SNPs (11 non-synonymous and 14 synonymous) showed statistically significant differences (P<0.05), and two SNPs, in position 1039 and 1041 of *cagE*, showed a highly significant association with cancer (p-value = 2.07×10^−6^), and the variant codon was located in the VirB3 homology domain of *Agrobacterium*. The results of this study may provide preliminary information to target antibiotic treatment to high-risk individuals, if effects of these variants are confirmed in further investigations.

## Introduction

*Helicobacter pylori* (HP) is one of the most common chronic bacterial infection in humans. It has been estimated that more than half of the adult population in the world is infected with this organism [1]. Among these, approximately 10–15% of the infected individuals are estimated to experience clinically adverse sequelae, including peptic ulcers, gastric adenocarcinoma and gastric mucosa-associated lymphoid tissue lymphoma (MALT) [2]. To date, despite extensive effort worldwide, what determines these variable clinical outcomes has not been fully elucidated, but believed to be combinations of environmental (e.g., smoking and diet) [3], host genetics and HP virulence factors [3], [4], [5]. Work by us [6] and others support that bacterial factors are likely to play the most decisive role [7], [8].

The best characterized HP virulence marker is the cytotoxin-associated gene pathogenicity island (cagPAI), a 40 kb region of chromosomal DNA encoding approximately 31 genes that forms a type IV secretion system (T4SS) to translocate bacterial products into the host cell. *cagA* resides within cagPAI and is responsible for most of the HP-associated malignant phenotypes: it triggers IL-8 secretion priming an inflammatory response, promotes cell proliferation, scattering and migration either through phosphorylation-dependent and independent mechanisms [9], [10]. The cagPAI is present in approximately 95% of East Asian isolates and it is less frequent in isolates from low risk Western countries [11], [12], [13].

Many of the cagA functions reside within a C-terminal tandemly arrayed repetitive motif containing the aminoacids Glu-Pro-Ile-Tyr-Ala (EPIYA motifs A, B, C and D). Strains harboring multiple copies of western type EPIYA-C or eastern type EPIYA-D are suggested to be more associated with gastric cancer and with an increased cagA *in vitro* activity [14], although this is controversial [15]. To date, despite the known variability in the N-terminal *cagA* gene and other cagPAI island genes, there has been very limited information concerning clinical relevance of genetic variants outside the EPIYAs. Thus, in this paper we seek to identify variants in the cagPAI genes *cagC* (*HP0546*), *cagE* (*HP0544*), *cagL* (*HP0539*), *cagV* (*HP0530*), *cagT* (*HP0533*), and *cag Gamma* (*HP0523*) genes, which have been designated as important functional components of model bacterial T4SS, and are known to be crucial for cagPAI translocation function or present extracellularly, suggesting a possible interactions with host cells; and in *cagA,* whose EPIYA region has been consistently shown to correlate with clinical outcome (gastric cancer) [16].

*cagA* status alone is not sufficient to predict clinical outcomes. Moreover there are indications that HP eradication reduces gastric cancer incidence only in individuals without precancerous lesions. The results of this study may provide valuable information to target antibiotic treatment to high risk individuals, if effects of these variants are confirmed in further investigations.

## Materials and Methods

### Ethics Statement

All participants signed an informed written consent. The study was approved by the ethical review boards of the institutions responsible for subject recruitment in each of the recruitment centres.

For Mexican samples, the study was approved by ethical committees of the Instituto Mexicano del Seguro Social (IMSS) and General Hospital of the Secretaria de Salud (SS), Mexico City, Mexico.

For Venezuelan samples, ethical clearance for the study was obtained from the International Agency for Research on Cancer (IARC) Ethical Committee in Lyon, France, and the Cancer Control Center in San Cristobal, Venezuela.

### Study population

#### Venezuela

We used 11 DNA samples from gastric biopsies from subjects affected with chronic gastritis without atrophy recruited in a chemoprevention trial in Venezuela [17], [18]. The study subjects (age 35-69) in this trial were recruited from participants in the Gastric Cancer Control Program of Tachira State, which was based on a gastric double contrast X-ray followed by a gastroscopic examination. Subjects with any cancer including gastric cancer, or with any other serious illnesses such as heart, lung, kidney or liver failure and pregnant women were not eligible. Seven gastric biopsies were taken from predefined sites, five for histological evaluation and two were frozen for *H pylori* DNA isolation or culture. Expert pathologists in neoplastic lesions of the stomach read histological slides.

#### Mexico

84 samples were from patients attending the Gastroenterology Unit of the México General Hospital (Secretaría de Salud) and the Oncology Hospital (Instituto Mexicano del Seguro Social), both hospitals in Mexico City. Thirty-five patients were affected with chronic gastritis and 49 with gastric cancer. Patients were older than 30 years, consulted because of gastroduodenal symptoms (General Hospital) or because of a probable gastric cancer (Oncology Hospital), and were programmed for endoscopy and biopsy for diagnostic purposes. Subjects who had previously received cancer treatment, were on antibiotics, anti-HP therapy or nonsteroidal anti-inflammatory drugs two weeks prior to the study, or had other severe chronic diseases were excluded. Gastric biopsy specimens were placed in sterile 0.9% saline solution, homogenized, and inoculated onto blood agar base (BBL, MD) plates supplemented with 5% sheep blood for HP culture. The plates were incubated at 37°C in a 9% CO_2_ atmosphere for up to 5 days. HP was identified by colony and microscopic morphology and by positive oxidase, catalase, and urease tests. From each primary growth, 7 to 10 single colonies each were isolated from the antrum and corpus and propagated on blood agar medium. For this study, we analyzed 43 samples from cultured strains and 41 directly from frozen biopsies.

The principal characteristics of the population are described in table 1.

**Table 1:** Characteristics of the populations and numbers of Mexican and Venezuelan samples for individual genes and regions of *cagA* analyzed.

		Mexican	Venezuelan	Total
	**Number of samples**	84	11	95
Diagnosis	**Cancer cases**	49	0	49
	**Gastritis cases**	35	11	46
Gender	**Female**	48	4	52
	**Male**	36	7	43
Median age	**Cancer cases**	58 (49–69)	-	58 (49–69)
(25%–75%)	**Gastritis cases**	44 (39.5–56.5)	54 (42.5–58.5)	46.5 (40–57.75)

Samples analyzed per gene				
	**CagA N terminal**	76	9	85
	**CagA middle region**	34	9	43
	**CagA EPIYA motif region**	60	10	70?
	**CagC**	35	9	44
	**CagE**	38	9	47
	**CagL**	33	0	33
	**CagT**	34	4	38
	**CagV**	18	9	27
	**Cag Gamma**	28	0	28

### DNA extraction

For Venezuelan and Mexican biopsy samples the DNA was extracted from frozen tissues using QIAamp DNA Micro Kit (Qiagen, Hilden, Germany) according to the manufacturer's instructions. For cultured strains DNA was purified using the guanidine thiocyanate-EDTA-Sarkosyl (GES) method [19].

### Primer design

We used alignments of HP sequences from public databases to identify sequences suitable for design of PCR primers. We limited our database searches to Western strains of HP, which are more likely to be similar to the strains found in our study samples. We designed tiled amplicons ranging in size from 312 to 876 bp. The average size of sequence reads with 454 sequencing technology is 450 bp, therefore forward reads and reverse reads overlap at least partially, thereby improving the reliability of the output. Five of the *cagA* amplimers used have been previously published [20], [21]. All primers used for *cagA*, *cagC, cagE, cagL, cagV, cagT* and *cag gamma* were first tested in PCR reactions on a small number of study samples (n = 16) and the amplified regions were sequenced with Sanger technology on the same samples to confirm the specificity of the amplification (see supplementary table S1 for primer sequences and PCR amplification conditions).

Furthermore, we used, as reference, three strains 26695 (NC_000195), J99 (NC_000921) and G27 (NC_0011333) whose genomes have been completely sequenced [22], [23], [24].

### 454 sequencing

Once PCR conditions were optimized, we resynthesized the same primers used for PCR with multiplex tags (used to identify sequences from each specific sample) and adaptors, and amplified the target regions using DNAs from samples. A second PCR was performed, using the tagged primers, in order to increase the amount of material. All PCR amplimers were then purified, quantified spectrophotometrically, and pooled in equimolar amounts.

Library generation for 454 FLX sequencing was carried out using the manufacturer's standard protocols (454 Life Sciences Corporation, Branford, CT, USA). In short, the manufacturer's adaptors required for processing and sequencing were added to the termini of each pool of tagged PCR products by ligation. Single molecules of the PCR products carrying the correct adaptors were hybridized to individual beads, clonally amplified in a subsequent emulsion PCR and each pool loaded onto a 1/16 of a picotiterplate for sequencing using the 454 GS FLX Titanium technology. After processing and base calling using the manufacturer's proprietary software (454 Life Sciences Corporation, Branford, CT, USA, Software version 2.0.00 October 2008) the resulting reads were sorted according to the pre-incorporated six base tags. Genomic sequence analysis by 454 technology was performed for these HP isolates with >200-fold average coverage (minimum 59x, maximum 580x). The resulting contigs were assembled using the gene sequence of HP strain 26695 [23] as a scaffold. We have not observed substantial differences in quality of the output between DNA from cultured strain and DNA from biopsies. In order to assess quality control of the data we compared 454 sequencing data of the reference strain 26695 and the published sequence in NCBI database (NC_000915); concordance was over >99%. We also sequenced 9 Venezuelan samples with the traditional Sanger sequencing method, observing a concordance >99% between methods.

### Sanger sequencing

The *cagA* N-terminal (630bp), C-terminal (position 2670-3100) and EPIYA motifs region as well as *cagL* gene were sequenced by the Sanger method. The sequencing reactions were performed using BigDyeR Terminator Cycle Kit (Applied Biosystems, Foster City, CA, USA) under thermal conditions as follow: 96°C for 2 min, and then 27 cycles at 96°C for 30 s, 54°C for 10 s and 60°C for 4 min. The reaction products were precipitated with 2-propanol, washed with 75% ethanol, diluted in 25 µl water and loaded onto an ABI prism 3100 Genetic analyzer (Applied Biosystems). Primary sequencing data were analyzed using a sequencing analysis program (Applied Biosystems).

### Bioinformatic and statistical methods

Raw sequences were automatically analyzed with the 454 software, and quality scores were assigned. The resulting sequencing output, from both 454 in sff format and Sanger in abi format, was analyzed with multiple sequence alignment software (e.g. the Geneious software platform: http://www.geneious.com/), which assembled all reads belonging to the same sample, then sequences of all the samples were aligned to a reference sequence and single nucleotide polymorphisms as well as small insertions and deletions were identified. To avoid potential artifacts from sequencing and to limit variants with clinically and statistically meaningful frequencies, we selected variants with unambiguous calls observed in at least 10% for synonymous (N = 175) and 20% for nonsynonymous (N = 206) variants of the originally tested samples and reference strains (Total 381).

SAS version 9.2 was used to estimate logit odds ratios (OR) and 95% confidence interval (CI) for gastric cancer associated with each variant as well as to calculate p-values for differences in variant frequencies between gastric cancer and gastritis by the Fisher's exact test (2-sided). Bonferroni correction was applied to compute p-values adjusted for multiple comparisons by dividing raw p-values with 381.

### Genetic variability in seven HP cagPAI genes

A summary of the genetic variability detected in the seven genes is reported in table 2. As expected, we observed a high degree of variability (computed as the number of sites showing a variant out of the total of sites in a gene), both at the DNA and amino acid level. The nucleotide variability ranged from 8.03% in *cagV* to 23.92% in *cagC*, while the amino acid variability interestingly ranged from 5.69% in *cagT*, showing the smallest degree of variation, to 31.01% in *cagA*.

**Table 2:** Summary of genetic variability in seven genes in HP cagPAI.

Gene	Number of nucleotides	Nucleotide differences^a^	Nucleotide differences (%)	Non-synonymous variants	Non-synonymous variants (%)	Number of amino acids	Amino acid differences^a^	Amino acid differences (%)
*cagA*	2670*	524	19.63%	333	63.55%	890*	276	31.01%
*cagC*	347	83	23.92%	32	38.55%	115	27	23.48%
*cagE*	2955	308	10.42%	68	22.08%	984	58	5.89%
*cagL*	714	74	10.36%	31	41.89%	237	29	12.24%
*cagT*	842	81	9.62%	18	22.22%	281	16	5.69%
*cagV*	759	61	8.03%	18	24.59%	253	15	5.93%
*cagGamma*	509	111	21.81%	40	36.04%	169	33	19.53%

a Computed as the number of sites showing a variant out of the total of sites (nucleotides or aminoacids) in a gene.

*single nucleotide polymorphisms in the EPIYA motif region are excluded

**Figure 1:**
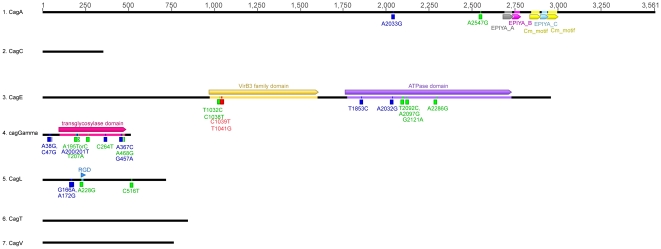
Summary of thirty-one SNPs (10 non-synonymous and 21 synonymous) showing statistically significant differences (P<0.05) between gastritis and gastric cancer cases. The color coding is as follows: SNPs in green are synonymous variants, SNPs in blue are not synonymous variants and SNPs in red are those with strong statistical significance (study-wise threshold of P = 1.31×10^−4^).

We compared the frequencies of the 381 selected genetic variants between gastric cancer and chronic gastritis cases. We then determined non-synonymous (table 3) and synonymous (table 4) variants that showed appreciable differences between gastritis and cancer cases, meeting one of the following criteria, (1) absolute variant frequency differed at least 25% between gastritis and cancer groups; (2) variant frequency in gastric cancer at least twice as high as in gastritis and (3) variant frequency in gastritis at least twice as high as in gastric cancer. Twenty-five SNPs (11 non-synonymous and 14 synonymous) reached statistically significant differences (p<0.05, figure 1), located in *cagA*, *cagE*, *caggamma* and *cagL*, whereas none were located in *cagC*, *cagT* or *cagV*. We then applied a study-wise threshold of p = 1.31×10^−4^ (0.05/381) adjusted for multiple comparisons, and only two SNPs, in position 1039 and 1041 in *cagE*, showed a p-value lower than this threshold. A SNP in the *cagE* gene (position 1905) shows a p value of 2.55×10^−4^ very close to the study-wise statistical significance.

**Table 3:** Non-synonymous variants with frequency greater than 10% in all samples and more of 25% difference in the prevalence between cancer and gastritis or the prevalence in one group at least twice as high as in the other group.

Genes	Position in 26695^a^	Position in contig	Nucleotide Change	Amino acid change	Gastric cancer cases/Cases of gastritis	Gastric cancer cases %	Cases of gastritis %	OR	(95% CI)^b^	Fisher P-value
*cagA*	193	193	G→A	G→R	44/40	13.6%	5.0%	3.00	(0.57–15.81)	0.269
*cagA*	334	334	A→G	K→E	45/39	15.6%	7.7%	2.21	(0.53–9.21)	0.327
*cagA*	344	344	A→G	Q→R	45/39	15.6%	7.7%	2.21	(0.53–9.21)	0.327
*cagA*	623	623	C→G	T→S	37/40	20.0%	8.1%	2.83	(0.69–11.63)	0.196
*cagA*	1696/1697	1696/1697	A→G/C→T	T→I/V	16/25	50.0%	80.0%	0.25	(0.06–1.00)	0.084
*cagA*	1814	1819	G→A	S→N	16/25	56.3%	84.0%	0.24	(0.06–1.05)	0.074
*cagA*	2033	2038	A→G	T→A	16/25	12.5%	48.0%	0.15	(0.36–0.88)	**0.041**
*cagA*	2341	2347	A→T	I→F	16/25	25.0%	12.0%	2.44	(0.47–12.78)	0.401
*cagC*	95	95	G→A	S→N	15/28	26.7%	10.7%	3.03	(0.58–15.88)	0.215
*cagC*	100	100	G→A	A→T	15/28	6.7%	17.9%	0.33	(0.03–3.11)	0.403
*cagC*	331	331	A→G	S→G	15/28	40.0%	14.3%	4.00	(0.91–17.55)	0.073
*cagE*	43	43	G→A	V→I	17/28	35.3%	64.3%	0.30	(0.09–1.07)	0.073
*cagE*	115	115	G→A	V→I	17/28	29.4%	14.3%	2.50	(0.57–11.06)	0.265
*cagE*	1039	1042	C→T	L→F	17/28	82.4%	10.7%	39.89	(6.90–219.11)	**2.07×10^−6^**
*cagE*	1661	1664	A→G	N→S	17/28	5.9%	17.9%	0.29	(0.03–2.70)	0.385
*cagE*	1853	1856	T→C	V→A	17/28	23.5%	64.3%	0.17	(0.04–0.67)	**0.013**
*cagE*	2032	2035	A→G	N→D	17/28	29.4%	67.9%	0.20	(0.05–0.73)	**0.016**
*cagE*	2509	2512	G→A	G→S	17/28	29.4%	14.3%	2.50	(0.57–11.05)	0.265
*cagL*	95	95	G→A	S→N	7/16	57.1%	18.8%	5.78	(0.82–40.76)	0.137
*cagL*	166	166	G→A	A→T	7/16	57.1%	6.3%	20.00	(1.63–247.98)	**0.017**
*cagL*	172	172	A→G	N→D	7/16	28.6%	81.3%	0.09	(0.01–0.73)	**0.026**
*cagL*	180	180	G→A	M→I	7/16	57.1%	25.0%	4.00	(0.61–26.12)	0.182
*cagL*	524	529	C→T	T→I	10/17	10.0%	23.5%	0.36	(0.03–3.79)	0.621
*cagGamma*	12	38	A→G	N→S	10/17	40.0%	5.9%	10.67	(0.98–115.68)	**0.047**
*cagGamma*	38	47	C→G	A→G	10/17	40.0%	5.9%	10.67	(0.98–115.68)	**0.047**
*cagGamma*	47	67	A→G	T→A	10/17	20.0%	5.9%	4.00	(0.31–51.03)	0.535
*cagGamma*	200/201	200/201	A→T	K→I	10/17	40.0%	88.2%	0.09	(0.01–0.62)	**0.025**
*cagGamma*	205/206	205/206	C→A/T→C	L→P/T	10/17	20.0%	52.9%	0.22	(0.03–1.37)	0.124
*cagGamma*	314	314	G→A	G→D	10/17	10.0%	41.2%	0.16	(0.02–1.55)	0.190
*cagGamma*	367	367	A→C	K→Q	10/17	40.0%	5.9%	10.67	(0.98–115.68)	**0.047**
*cagGamma*	457	457	G→A	A→T	9/16	22.2%	68.8%	0.13	(0.02–0.86)	**0.041**

a Reference strain 26695 (reference in GenBank: NC_000915).

b OR =  odds ratio; CI = confidence interval

**Table 4:** Synonymous variants with a frequency greater than 20% in all samples and more of 25% difference in the prevalence between cancer and gastritis or the prevalence in one group at least twice as high as in the other group.

Genes	Position in 26695^a^	Position in contig	Nucleotide change	Gastric cancer cases/Cases of gastritis	Gastric cancer cases %	Cases of gastritis %	OR	(95% CI)^b^	Fisher P-value
*cagA*	858	861	T→C	16/25	12.5%	40.0%	0.21	(0.04–1.15)	0.084
*cagA*	870	873	T→C	16/25	62.5%	36.0%	2.96	(0.81–10.88)	0.120
*cagA*	1743	1746	C→T	16/25	50.0%	76.0%	0.32	(0.08–1.21)	0.105
*cagA*	2547	2553	A→G	16/25	31.3%	68.0%	0.21	(0.06–0.83)	**0.029**
*cagC*	252	252	C→T	15/28	60.0%	32.1%	3.17	(0.86–11.65)	0.109
*cagC*	288	288	T→C	15/28	6.7%	32.1%	0.15	(0.02–1.33)	0.127
*cagE*	69	69	C→G	17/28	52.9%	78.6%	0.31	(0.08–1.14)	0.101
*cagE*	72	72	A→G	17/28	52.9%	78.6%	0.31	(0.08–1.14)	0.101
*cagE*	462	462	G→A	17/28	41.2%	67.9%	0.33	(0.10–1.16)	0.121
*cagE*	1011	1011	C→T	17/28	70.6%	42.9%	3.20	(0.89–11.56)	0.123
*cagE*	1032	1035	T→C	17/28	11.8%	42.9%	0.18	(0.03–0.93)	**0.046**
*cagE*	1038	1041	C→T	17/28	94.1%	64.3%	8.89	(1.02–77.32)	**0.033**
*cagE*	1041	1044	T→G	17/28	82.4%	10.7%	38.89	(6.90–219.11)	**2.067×10^−6^**
*cagE*	1164	1167	T→C	17/28	11.8%	25.0%	0.40	(0.07–2.20)	0.447
*cagE*	1905	1908	T→C	17/28	29.4%	85.7%	0.07	(0.02–0.31)	**2.55×10^−4^**
*cagE*	2058	2061	A→G	17/28	35.3%	64.3%	0.30	(0.09–1.07)	0.073
*cagE*	2092	2095	T→C	17/28	35.3%	71.4%	0.22	(0.06–0.79)	**0.029**
*cagE*	2097	2100	A→G	17/28	35.3%	71.4%	0.22	(0.06–0.79)	**0.029**
*cagE*	2121	2124	G→A	17/28	47.1%	89.3%	0.11	(0.02–0.49)	**0.004**
*cagE*	2286	2289	A→G	17/28	52.9%	89.3%	0.14	(0.03–0.62)	**0.011**
*cagL*	228	228	A→G	7/16	71.4%	12.5%	17.50	(1.82–159.53)	**0.011**
*cagL*	375	379	G→A	7/16	27.3%	57.9%	0.27	(0.05–1.36)	0.142
*cagL*	477	482	T→C	10/17	10.0%	23.5%	0.36	(0.03–3.79)	0.621
*cagL*	516	521	C→T	10/17	90.0%	47.1%	10.13	(1.05–98.49)	**0.042**
*cagT*	336	339	T→C	14/23	21.4%	47.8%	0.30	(0.07–1.36)	0.166
*cagT*	339	342	A→G	14/23	21.4%	43.5%	0.35	(0.08–1.62)	0.288
*cagV*	243	243	C→A	8/18	62.5%	22.2%	5.83	(0.95–35.72)	0.078
*cagV*	306	306	A→C	8/18	87.5%	55.6%	5.60	(0.57–55.43)	0.190
*cagGamma*	150	150	C→T	10/17	60.0%	88.2%	0.20	(0.03–1.40)	0.154
*cagGamma*	195	195	A→C/T	10/17	40.0%	88.2%	0.09	(0.01–0.62)	**0.025**
*cagGamma*	207	207	T→A	10/17	40.0%	88.2%	0.09	(0.01–0.62)	**0.025**
*cagGamma*	264	264	C→T	10/17	40.0%	100.0%	0.02	(0.00–0.42)	**0.001**
*cagGamma*	468	468	A→G	9/16	88.9%	43.8%	10.29	(1.03–102.75)	**0.041**

a Reference strain 26695 (reference in GenBank: NC_000915).

b OR =  odds ratio; CI = confidence interval

### *cagA* polymorphisms and EPIYA types

**Figure 2:**
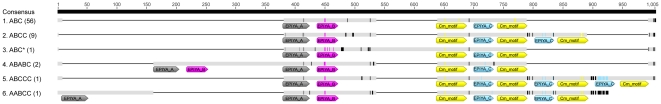
Scheme of the alignment of the EPIYA motifs with number of observations in Mexican and Venezuelan populations.

The C-terminal region (positions 2670 to 3100) was highly variable in the clinical isolates according to the pattern of the EPIYA motifs (figure 2). We observed 524 polymorphic sites of which we analyzed 148 selected with the criteria previously described (the complete catalog of *cagA* SNPs is shown in supplementary file S1). Interestingly, two SNPs show a different recurrence between gastritis and cancer cases with p<0.05, even if they were not considered statistical significant due to the large number of tests; one is a non-synonymous SNPs (A2033G determine an amino acid change T/A, see table 3) and one synonymous SNPs (A2547G, see table 4).

The analysis of the EPIYA region confirmed that all sequences were of the Western type cagA, i.e., ABC (82%), ABCC (13%), ABABC (3%), AABCC (1%) and ABCCC (1%). We did not observe a different distribution of these cagA types between the cancer and gastritis cases (p = 0.2342), detailed results are shown in table 5 and figure 2. We observed 3 variants of the EPIYA motif: one gastritis sample had EPIYV in an A motif, 50% of B motifs showed the EPIYT variant (no statistical different distribution in cancer and gastritis case) and one C motif of a cancer case showed a EPLYA variant.

**Table 5:** Patterns of *cagA* EPIYA motifs in cancer and gastritis samples.

EPIYA motif	Number of samples (%)	Cancer (%)	Gastritis (%)
AABCC	1 (1.43%)	0 (0%)	1 (3.70%)
ABABC	2 (2.86%)	1 (2.33%)	1 (3.70%)
ABC	57 (81.43%)	38 (88.37%)	19 (70.37%)
ABCC	9 (12.86%)	4 (9.30%)	5 (18.52%)
ABCCC	1 (1.43%)	0 (0%)	1 (3.70%)
Total	70 (100%)	43 (100%)	27 (100%)

P = 0.2342 (Chi square test).

### Results in the other *cag*PAI genes

Genetic variability of *cagC cagE*, *cagT, cagV* and *cag Gamma* was assessed by 454 sequencing and of c*agL* by Sanger sequencing. The complete catalog of polymorphisms observed in these seven genes is shown in supplementary file S1.

In the *cagC* gene we detected 83 SNPs, 25 of which were selected as described above. None of these SNPs showed a differential distribution between gastritis and cancer cases.

In the *cagE* gene we have catalogued 308 polymorphic sites, 97 of these polymorphisms were analyzed. C1039T and T1041G showed a statistically significant different recurrence between gastritis and cancer cases with p = 9.97×10^−6^. Furthermore another SNP T1905C showed a different recurrence with a p value of 2.55×10^−4^, which is very close to the study-wise threshold. Nine other SNPs show a different recurrence between gastritis and cancer cases with p<0.05, six synonymous polymorphisms (T1032C, C1038T, T2092C, A2097G, G2121A and A2286G) and 3 non-synonymous variants: A76C (aminoacidic change from lysine to glutamine), T1853C (aminoacidic change from valine to alanine) and A2032G (aminoacidic change from asparagine to aspartic acid).

The C1039T variant, when analyzed as single change, predicts an amino acid change of lysine to phenilalanine (codon change of CTT to TTT), whereas the SNP T1041G lies at the third position of the same codon and if analyzed as single change it predicted a synonymous variant (codon change CTT to CTG). However, in all the samples that we have analyzed the two variant alleles were observed together, therefore we observed only two variant codons (CTT and TTG) which encode for the same amino acid, lysine. The T1905C polymorphism is a synonymous variant at the third position of the GTT codon (variant codon GTC), which codes for Valine.

In the *cagL* gene we observed 74 polymorphisms and 24 of which were analyzed, 4 showed a differential distribution between cases of cancer and gastritis (P<0.05). Two of them were non-synonymous: G166A (aminoacidic change of alanine to threonine) and A172G (aminoacidic change of asparagine to aspartic acid) and two synonymous: (A228G and C516T).

In the *cagT* gene we analyzed 23 of the 81 polymorphisms observed, while in the *cagV* gene we analyzed 11 of the 61 polymorphisms, and in both genes none of the polymorphism showed a differential distribution between gastritis and cancer cases.

In the *cag Gamma* gene we observed 111 polymorphisms, 53 of which were further analyzed and 4 synonymous (A195TorC, T207A, C264T and A468G) and five non-synonymous (A38G, C47G, A200/201T, A367C and G457A) showed a p<0.05 for the differential distribution between gastritis and cancer cases (figure 1).

## Discussion

Since its discovery in 1996 [25], the cagPAI, which harbors the virulence genes of HP, has probably been the most intensively studied part of the HP genome. The type IV secretion system encodes proteins, which form a needle-like structure connecting HP to the cytoplasm of the epithelial gastric cell to inject the oncogenic CagA protein and peptidoglycans. Components of this structure include a) pilus components, CagC (homologue of the *Agrobacterium tumefaciens* VirB2) forming the main extracellular structure, to which the tip CagL is attached to interact with β-1 integrin; b) core complex proteins, CagW (VirB6), CagT (VirB7), CagV (VirB8), CagX (VirB9) and CagY (VirB10) which form the inner core of the pilus; c) energetic factors Cagβ (VirD4), Cagα (VirB11) and CagE (VirB3/VirB4), ATPases supplying energy for the system to work [26]. In this study we have sequenced *cagC* (*HP0546*), *cagL* (*HP0539*) from the pilus, *cagV* (*HP0530*), *cagT* (*HP0533*), and *cag Gamma* (*HP0523*) from the core complex, and *cagE* (*HP0544*) from the energy supply enzymes from HP strains isolated from gastritis and gastric cancer patients. These genes were chosen because their products are known to be essential for T4SS function and some are presented extracellularly by *H. pylori* (CagA, CagL, CagC), suggesting possible interactions with the host cell [27].

We found the smallest variation, both at nucleotide and aminoacid level, in the inner core T4SS components (CagT, CagV, and CagE; 5.7%, 5.9% and 5.9% aminoacid variation, respectively), and the largest variation in the exposed components: integrin binding protein CagL, extracellular pilus main component CagC and secreted protein CagA (12.2%, 23.5% and 31%, aminoacid variation, respectively). These results support that genetic variation in cagPAI components is mainly influenced by their localization in the T4SS, with higher variation in proteins exposed in the bacterial surface, perhaps as a response to immunological pressure. Interestingly, Cag Gamma was an exception (19.5% aminoacid variation), this protein has been proposed to reside within the HP periplasm where it acts as a peptidoglycan hydrolase, piercing the HP outer membrane and thus helping to expose the T4SS pilus to the external medium [28]. It is possible that Cag Gamma fulfills this function also as a structural component of the exposed pilus, where it could also act over the host cell membrane.

Studies from Africa [21], Italy [14], USA [8] and Brazil [29] have suggested an association between increased number of EPIYA C motifs and HP associated diseases. Furthermore, Sicinschi et al. [30] observed an association between increased EPIYA C segments and the presence of gastric precancerous lesions. In contrast, studies in Colombia [8], [31], Mexico (J. Torres, personal communication), and Korea [32] have not found such an association. In our study, over 80% of all samples from Mexico and Venezuela were of the type ABC, and no association was evident between gastric cancer progression and higher number of EPIYA C motifs. Moreover, recent studies [15] have shown the importance of point variations in EPIYA B motif for activity on epithelial cells, we observed four non synonymous variations in this motif, but these polymorphisms did not show an association with gastric cancer.

Recent studies have reported important pro-inflammatory and pro-oncogenic activities of CagA that are independent of the EPIYA motifs and which might be as important for disease [30]; these findings could explain the lack of association of C motifs with cancer reported here and in previous studies. The C terminal of CagA protein also contains the C-MET motif which has been proposed to have several functions: mediate CagA multimerization and membrane targeting [33], [34], interact with the kinase Par1b/MARK2 [35], and all these activities are CagA-phosphorylation independent [36]. However, in our study we did not find significant differences between gastritis and gastric cancer, either in sequence or in the number of multimerization motifs.

The C-terminal and N-terminal domains of CagA are both required to exploit the full activity of the protein, although they have distinct functions. Recently it has been shown that the N terminus of CagA interacts with the tumor suppressor apoptosis-stimulating protein of p53 (ASPP2) [37].

The presence of all these interactions between CagA and bacterial and human proteins suggest that it could be very difficult for the bacterium to maintain the full range of biological activity in the presence of high level of mutations, most of which presumably lead to loss or attenuation of function.

Although *cagA* is the best established cagPAI virulence marker, *cagA* status alone is not sufficient to predict clinical outcomes in high risk populations where the majority of HP are *cagA*-positive strains. In this context, identification of new HP molecular virulence markers to predict gastric cancer risk will be very important. Recent progress in genotyping methodology enables us to use DNA from gastric biopsies to study HP sequence microvariabilities, which have been almost exclusively studied in cultured strains. Genotyping of a higher number of gastric samples allows us to expand cagPAI genetic variant detection to other potentially important T4SS genes. This is relevant since earlier studies have focused mainly on *cagA* and the utility of other cagPAI genes as markers for disease risk is scarcely studied. Few studies have looked for the association of presence of cagPAI genes and disease, and none have studied polymorphisms in cagPAI genes, other than *cagA.*

*cagE* is a unique gene that encodes two T4SS components, VirB3 (N-terminal) and B4 (C-terminal) as a fusion protein[38], and B4 is the largest ATPase among several T4SS components. It generates energy for the secretion process, thus is required for substrate translocation [39] and interacts with many other T4SS proteins including VirB2 [40]. Despite its relatively inner localization, its pivotal role in IL-8 induction has been well documented [41], [42], [43]. Interestingly, we observed a strong association between two SNPs (C1039T and T1041G) of the *cagE* and gastric cancer, a finding not reported previously. These SNPs are at the position one and three of the same codon and we always observed these two variant codons (CTT and TTG) which codify for the same amino acid, lysine. This variant codon is located in the homology domain with VirB3 of *Agrobacterium*. The second strongest association was detected in another synonymous SNP in position 1905 and in this case the two possible codons were GTT and GTC, which code for valine. It has been known for a long time that alternative synonymous codons are not used with equal frequencies and patterns of codon usage vary among species [44]. Codon usage is more biased in genes expressed at higher levels [45], [46]. The use of optimal codons allows for more efficient use of ribosomes and leads to faster growth rate [47]. Although the genome of HP has been reported to contain no codon bias for highly expressed genes [48], Kloster and Tang [49] identified a bias in the expression level of genes where TTG codon is preferred over the CTT codon, as well as of the GTC codon over the GTT. Therefore, based on these data we could speculate that difference in codon usage may have an impact on the level of expression of a gene with a strong functional relevance for the T4SS secretion system such as *cagE*.

CagL is a specialized pilus protein that binds to and activates integrin α5β1 receptor on gastric epithelial cells primarily through its arginine-glycine-aspartate (RGD) motif, guiding proper positioning of the T4SS and facilitating translocation of cagA [9], [50]. CagL also activates the host cell kinases focal adhesion kinase (FAK) and Src to ensure CagA phosphorylation at the site of injection, whereas β1 integrin is required for CagA-induced host cell motility and elongation [51]. CagL may also be responsible for HP-induced hypochlorhydria through activation of a disintegrin and metalloprotease 17 and of NFκB [52]. Of the two *cagL* SNPs we found associated with gastric cancer, the A172G SNP (N58D) is in the same position in which Yeh et al [53] have demonstrated that the concurrent presence of tyrosine in amino acid position 58 and glutamic acid in position 59 (Y58E59) compared with the combination aspartic acid (D58) and Lysine (K59), induces more efficiently a corpus shift of gastric integrin α5β1 which has been related with gastric carcinogenesis. We did not observe the tyrosine (Y) amino acid in position 58 in any sample, although we found that carriers of aspartic acid (D) at this position are at lower risk of gastric cancer in comparison with the asparagine (N) carriers. Furthermore, we observed the polymorphism in amino acid 59 at lower frequency and without any difference between cancer and gastritis samples.

In previous studies [54] sequence analysis of *cagGamma* gene showed that it harbors a typical SLT catalytic domain between residues 33 and 165, whose “ES” and “AVGAY” motifs were highly conserved among the ortholog enzymes. We observed five nonsynonymous variants with a different distribution in cancer and gastritis cases. Three of those map in the catalytic domain, nevertheless none of them is located in the most conserved parts of the domain.

Although this study has limitations in sample size, it is the largest up to now in terms of the number of cagPAI genes studied for a deep sequence analyses. A few samples were lost because of failure in PCR amplification, which may be due to microvariabilities of HP sequence. Loss due to poor quality score from 454 sequencing are relatively limited and derive from low efficiency of original PCR, which did not allow a equimolar pooling. We limited statistical analysis to variants with very high frequencies to avoid potential artifacts from high throughput sequencing; consequently, it is possible that rare variants with significant effect on T4SS functions are not represented in this study. Still, it is unlikely that such variants account for a sizable fraction of patients who develop HP associated gastric cancer.

Our study was limited to Venezuelan and Mexican populations and the results cannot necessarily be extrapolated to other populations. Particularly we did not observe any Eastern strains and we cannot analyze the impact of these polymorphisms on such different genetic background. In fact, analysis of EPIYA motifs between Venezuelan and Mexican strains showed a highly similar distributions in the two populations, which share similar genetic background. Furthermore the exclusion of Venezuelan samples did not change the results on *cagE* position 1039/1041 polymorphisms, which remained highly statistically significant (Fisher p-value  =  5.04×10^−5^). In addition, because the numbers of gastric cancer cases assessed for individual cag genes were very limited (generally <20), it was not statistically appropriate to include extra covariates such as age. Age is an important determinant of gastric cancer risk and may exert the cohort effect on the type of strains acquired. However, when we examined the effects of age on the prevalence of specific cag A/E/L and gamma SNPs associated with gastric cancer risk, the association was not statistically significant with p-values ranging from 0.130-0.930. Thus, we believe the effect of confounding from age is very small if any.

It is also possible that strains from cancer cases may simply represent the ones that can survive in a cancer environment. As gastric adenocarcinoma is known to arise from several stages of premalignant lesions, i.e., chronic gastritis, atrophic gastritis, intestinal metaplasia (IM) and dysplasia, induced by HP infection [2], [55], these associations need to be confirmed using samples from various stages of premalignant lesions in future. Furthermore, functional tests are warranted to clarify the role of these variants in the pathogenicity and address future studies towards more targeted preventive interventions. We are planning to test strains with variants in *cagE* and *cagL* in cultured epithelial cells to elucidate if translocation and morphology effects are altered, and in a mouse model to study their ability to induce gastric tumors.

Although *cagA* is the best established HP virulence marker, *cagA* status alone is not sufficient to predict clinical outcomes in high risk populations where the majority of HP are *cagA*-positive strains. In this study we show that polymorphisms in genes coding for energy-supply protein CagE and for the β-1 integrin recognizing CagL may also affect virulence, most probably because they are necessary for a functional secretory system. We also document that genetic variation is higher for genes encoding proteins exposed to the host milieu, probably because of a positive selection exerted by the inflammatory and immune response of the host.

## Supporting Information

Table S1 Primers used for PCR amplification and nucleotide sequencing.
(DOC)

File S1 Complete catalog of single nucleotide polymorphisms in seven cagPai genes. Positions refer to 26695 HP strain.
(XLS)
