# Supplementary material for: Variations in Helicobacter pylori Cytotoxin-Associated Genes and Their Influence in Progression to Gastric Cancer: Implications for Prevention
Source: PLoS One. 2012 Jan 3;7(1):e29605. doi: 10.1371/journal.pone.0029605 (PMC3250449; doi:10.1371/journal.pone.0029605)
Supplement: Table S1 — Primers used for PCR amplification and nucleotide sequencing. (DOC) [file pone.0029605.s001.doc]

**Supplementary table S1**. Primers used for PCR amplification and nucleotide sequencing.

| **Primer name** | Orientation (ref) | Sequence | Amplimer length | Position* | Protocol** |
| --- | --- | --- | --- | --- | --- |
| **CagA** |  |  |  |  |  |
| cagA 1 | Fow | GAGAAACAATGACTAACGAAAC | 661 | -8 | 3 step 55°C |
|  | Rev (1) | GAGAGAAAAATATCCAACCAATC |  | 653 |  |
| CagA11F | Fow (1) | TTCATGGGCGTGTTTGATG | 854 | 558 | TD60-50 |
|  | Rev (1) | ATAATCTTTGAGAGTGTAGCTC |  | 1412 |  |
| CagA12F | Fow (1) | GCTTATTTAGACGCCCTAG | 699 | 1314 | TD60-50 |
|  | Rev (1) | GTAAGCGATTGCTCTTGC |  | 2013 |  |
| cagA 4 | Fow | TTTCAAATACACCAACGCC | 552 | 1529 | 3 step 56°C |
|  | Rev | TTTTCAAGTTTATCAGACAATTC |  | 2081 |  |
| CagA 5 | Fow | GAATTGTCTGATAAACTTGAAAA | 707 | 2058 | TD60-50 |
|  | Rev | GCAACTTGAGTATAAATGGG |  | 2765 |  |
| cagA15 | Fow (2) | GGAATTGTCTGATAAACTTG | 594 | 2057 | 3 step 50°C |
|  | Rev (2) | CCATTATTGTTATTGTTATTG |  | 2651 |  |
| cagA16 | Fow (2) | GGAACCCTAGTCGGTAATG | 446 | 2659 | 3 step 50°C |
|  | Rev (2) | ATCTTTGAGCTTGTCTATCG |  | 3105 |  |
| **CagC** |  |  |  |  |  |
| CagC | Fow | TCAATTTGAAAGGAAACG |  | -12 | 3 step 52°C |
|  | Rev | CTAACCCATACGAACTGAAA | 349 | 248+89 |  |
| **CagE** |  |  |  |  |  |
| CagE1 | Fow | ATACGATTACATGTGAAGCG | 341 | -40 | 3 step 52°C |
|  | Rev | TCTTGAATCCAAGAGCTACA |  | 301 |  |
| CagE2 | Fow | GCGATTGTTATTGTGCTTGTAG | 332 | 264 | 3 step 54°C |
|  | Rev | TTGGAAGTGGTTAAAAAATCAAT |  | 596 |  |
| CagE3 | Fow | CTCTTTTAGGCACAATCAAA | 521 | 544 | 3 step 52°C |
|  | Rev | AAAAGGGGCTTCATTCATT |  | 1065 |  |
| CagE4 | Fow | AATGAATGAAGCCCCTTTT | 384 | 1046 | 3 step 54°C |
|  | Rev | CCATAACTGCCTAGCGTAAT |  | 1430 |  |
| CagE5 | Fow | ATTACGCTAGGCAGTTATGG | 387 | 1410 | 3 step 56°C |
|  | Rev | GGTTGAGCCAAGTATCAAAG |  | 1797 |  |
| CagE6 | Fow | GGACACACTTTGATACTTGG | 560 | 1770 | 3 step 52°C |
|  | Rev | TCAAAAAGCCATTGAAATTC |  | 2330 |  |
| CagE7 | Fow | GAATTTCAATGGCTTTTTGA | 344 | 2310 | TD62-52 |
|  | Rev | TTTGTAGGGCATTGTTCTCT |  | 2654 |  |
| CagE8 | Fow | TTGTAAGAGACATGCTAAAAAC | 490 | 2530 | 3 step 52°C |
|  | Rev | CCAAAATCGTCAAGACATCA |  | 3020 |  |
| **CagL** |  |  |  |  |  |
| CagL1 | Fow | ATCCCAAAGGTTAGAAATCA |  | 740+15 | TD60-50 |
|  | Rev | ACTTATATGTCTAGCCCTGAAC |  | 325 |  |
| CagL2 | Fow | GCTCAGCAGTATTTTGGTCT |  | 356 | 3 step 54°C |
|  | Rev | AACGACAGCAAGAAACATTG |  | -42 |  |
| **CagT** |  |  |  |  |  |
| cagT1 | Fow | GAACCATGTTTATACGCCTG |  | 95 | 3 step 56°C |
|  | Rev | CTCGCTATCATCACCACACC |  | 407 |  |
| cagT2 | Fow | GGTGTGGTGATGATAGCGAG |  | 387 | 3 step 56°C |
|  | Rev | TCACTTACCACTGAGCAAACT |  | 843 |  |
| **CagV** |  |  |  |  |  |
| CagV | Fow | GGGTGTGATAGCCCTTGATA |  | 50 | 3 step 54°C |
|  | Rev | TATCATCAATCCTTTTGGCT |  | 649 |  |
| **HP0523** |  |  |  |  |  |
| HP0523 | Fow | GTTCAAAATCTGCTCTCACC |  | -96 | TD60-50 |
|  | Rev | TAGAAATGGCAATAGGCG |  | 521 |  |

* positions are referred to gene sequence in 26695 strain

** **protocol**:

10ngDNA, 10 pmol primer (each) MgCl 2mM, Dntp 0.2mM, 0.125 U taq polymerase per reaction

**3 step**

step 1:95°C 1min

step 2: annealing temperature as reported in the table per1min

step 3:72 °C 90sec

step 4:repeat from step1 to step 3 per 39 cycles

step5:72 °C per 5 min.

**touch-down protocol (TD 60-50):**

step 1:95°C 1min

step 2:60 °C 1min decrease 1 °C per cycle

step 3:72 °C 90sec

step 4:repeat from step1 to step 3 per 10 cycles

step 5:95°C 1min

step 6: 50 per1min

step 7:72 °C 90sec

step 8:repeat from step5 to step 7 per 29 cycles

step9:72 °C per 5 min.

**touch-down protocol (TD 62-52):**

step 1:95°C 1min

step 2:62 °C 1min decrease 1 °C per cycle

step 3:72 °C 90sec

step 4:repeat from step1 to step 3 per 10 cycles

step 5:95°C 1min

step 6: 52 per1min

step 7:72 °C 90sec

step 8:repeat from step5 to step 7 per 29 cycles

step9:72 °C per 5 min.

1) Argent Clin Cancer Res 2227 2008;14(7) April 1, 2008

2) Rudi 1998, JOURNAL OF CLINICAL MICROBIOLOGY,Apr. 1998, p. 944–948
